# A Contextual Nutrition Education Program Improves Nutrition Knowledge and Attitudes of South African Teachers and Learners

**DOI:** 10.3389/fpubh.2019.00258

**Published:** 2019-09-18

**Authors:** Mojisola Deborah Kupolati, Una E. MacIntyre, Gerda J. Gericke, Piet Becker

**Affiliations:** ^1^Human Nutrition Department, University of Pretoria, Pretoria, South Africa; ^2^Research Office, Faculty of Health Sciences, University of Pretoria, Pretoria, South Africa

**Keywords:** contextual nutrition education program, nutrition knowledge, teachers, learners, impact evaluation, attitudes, practices

## Abstract

**Background:** Evaluating the impact of a nutrition education program could provide insight into the effectiveness of an intervention. Researchers tested the hypothesis that a theory-based contextual nutrition education program (NEP) would improve the nutrition knowledge, attitudes, and dietary practices (KAP) of teachers and learners.

**Methods:** Twenty three teachers who taught nutrition in Grades 4–7 (treatment school, *n* = 12) and 681 learners (treatment school, *n* = 350) participated in the study. In this quasi-experimental study, two primary schools were randomly selected to implement a contextual NEP. The nutrition KAP were assessed using previously validated questionnaires. The treatment school teachers taught nutrition using a developed nutrition education manual, while the control school teachers taught nutrition in the usual manner. Random effects Generalized Least Squares regression estimated the difference in the teachers' and learners' KAP for the treatment and control schools; *p* = 0.025 for a one-tailed test.

**Results:** At post-implementation, the treatment school teachers' had higher total nutrition knowledge mean score (85.5% ± 8.2, *p* = 0.003) compared to the control school. Within the treatment school, total nutrition knowledge mean score of the teachers improved by 14.1%, *p* ≤ 0.001. Learners in the treatment school had higher total nutrition knowledge (53.2% ± 16.9, *p* = 0.002) and nutrition attitude (63.9% ± 19.7, *p* = 0.001) scores compared to learners in the control school. Within the treatment school, learners' total nutrition knowledge and nutrition attitudes scores increased by 4.9%, *p* ≤ 0.001 and 6.9%, *p* ≤ 0.001, respectively. The dietary practices of the teachers and the learners, and the nutrition attitudes of the teachers in the treatment school showed no significant within school improvement or in comparison with the control school (*p* > 0.025).

**Conclusions:** The NEP led to the improvement in the teachers' and the learners' nutrition knowledge and the learners' nutrition attitudes. However, no significant improvement in the dietary practices of either teachers or learners was found.

## Introduction

School-based nutrition education (NE) has been widely used to address nutrition and health-promotion initiatives for learners, teachers, and school staff (1). Children with unhealthy eating behaviors may become malnourished, and may develop non-communicable diseases (NCDs) such as hypertension, coronary heart disease, diabetes, and obesity later in life (1, 2). Effective school-based NE is a practical approach for reducing malnutrition and can increase the Gross Domestic Product (GDP) of a country by 2–3% (2). School-based NE contributes to healthy growth and development, increases attention span, and improves children's learning capacity leading to scholarly achievement and increased future earnings (2, 3). A global review of NE revealed that school-based NE interventions implemented by trained teachers improved behavioral outcomes of learners (3). Teachers play an important role in school-based nutrition education, and they should be equipped to influence the dietary behaviors of learners positively (4, 5). One way of empowering teachers is through a NE intervention.

School-based NE often includes components such as curriculum enhancement, parental involvement, hands-on activities, gardening, physical activities, and card or computer games (6–8). When enriching nutrition curricula, input from teachers may increase the feasibility of a program, and increase teachers' commitment to implementation (9). Nutrition curricula should also include experiential activities that stimulate new learning in children. Nutrition education programs that include hands-on activities are known to improve dietary behavior among learners from economically disadvantaged backgrounds (10–12).

Learners from low economic settings have unique issues, which need to be considered when providing NE. One such consideration is a limited diversity of foods. There is sometimes a misperception about indigenous foods resulting in the limited use of otherwise healthy choices of locally available foods (13). Therefore, it is necessary to make NE contextual by integrating assessed needs and involving community participation (14, 15). School NE in a low economic environment needs to focus on equipping learners to be future parents who could take responsibility for the nutritional well-being of themselves and that of their families. Learners cannot be expected to apply information that is not pertinent to their low economic background (16). Furthermore, it will be beneficial to introduce NE in the early years of schooling in low economic communities so that learners who drop out of school early to start raising a family, could benefit from NE through appropriate nutrition knowledge (17).

Many NE intervention studies, especially among populations in low economic settings, lack an impact assessment (1, 18). Impact studies assessing the effectiveness of NE interventions are thus needed to estimate change, especially where interventions aim to address existing NE concerns (19–21). Impact assessments measure how target behaviors change in relation to interventions. Impact evaluations are most appropriate when the issue of cause and effect is of importance to an intervention (22). A sound NE impact evaluation comprises an intervention design that can be evaluated, including impact measures that align with the type of NE intervention and the impact data collected after implementation (22).

Previous NE intervention studies involving teachers in South Africa revealed an improvement in teachers' and learners' nutrition knowledge and self-efficacy (23–25). Learners from poor socio-economic settings have specific NE concerns, such as unhealthy eating among learners, a need for an appropriate educational strategy and a need for an effective approach for communicating nutrition messages (20, 21, 26). This study implemented a context-specific nutrition education program (NEP) developed for Grade 4–7 teachers and tested the hypothesis that the NEP would significantly improve the nutrition knowledge, attitudes, and dietary practices (KAP) of the teachers and the learners in the treatment school.

## Materials and Methods

### Study Setting and Participants

Schools in the Bronkhorstspruit district, Gauteng Province, South Africa participated in the study. In South Africa, primary school children are aged from 6 to 13 years for Grade 1 through Grade 7 (27). The schools were recommended by the Gauteng Department of Basic Education (DoBE) and were in quintile 2 of the national poverty classification system. The quintile system ranks public schools in South Africa into five categories based on a poverty index. Quintiles 1, 2, and 3 are the poorest quintiles. They are no-fee schools and receive more state support, including financial support and school meals, than the fee-paying schools in quintiles 4 and 5 (28).

Bronkhorstspruit district has 13 public primary schools. Twelve schools agreed to participate and were divided into two clusters; small schools (*n* = 5, learner enrolment <1,000) and large schools (*n* = 7, learner enrolment ≥1,000). For this study, we selected schools in the large cluster due to the large number of teachers and learners. Two schools were randomly chosen, and designated as treatment and control schools by an independent person who tossed a coin. In order to avoid a spill over effect, we ensured the treatment and control schools were not within proximity; 6 km apart. Also, the control school had no access to any of the nutrition education materials used by the treatment school. Furthermore, no potential confounding variables were observed. Within each school, we selected a convenience sample of teachers of Life Skills (LS) and Natural Science and Technology (NST) in Grades 4–7, and learners in Grades 5 and 6. Participants included all the teachers of LS and NST for Grades 5 and 6, who signed informed consent forms, and learners (Grades 5 and 6) with parental consent, and who themselves gave assent and were present on the days of data collection. Learners in Grades 5 and 6 were chosen because the highest number of nutrition topics is taught in these grades as outlined in the DoBE curriculum.

### Study Design

A quasi-experimental design that involved two schools, randomly allocated as a treatment and control school, was employed. A developed contextual NEP was implemented in the treatment school. The impact of the intervention on the participants' nutrition KAP was evaluated.

### Intervention

The researchers developed a set of nutrition education materials, comprising a teacher's manual, picture book, learner's workbooks, and posters (29). Selected constructs of the Social cognitive theory (SCT) (30) and the Meaningful learning model (MLM) (31), in combination with the results of a prior needs assessment (32–34), were used to explain the nutrition topics in the existing curriculum of the DoBE.

**Figure 1:**
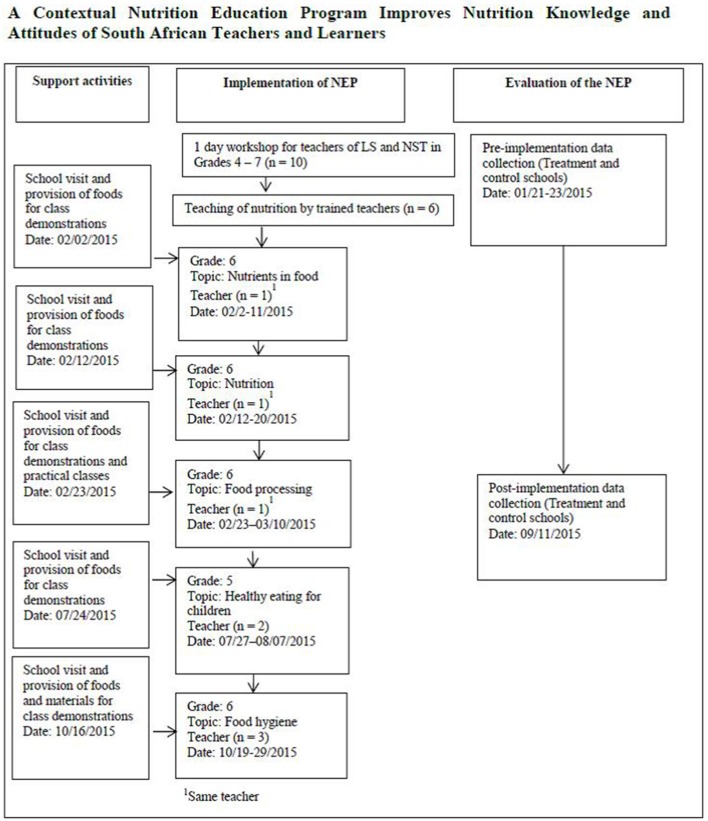
Implementation of NEP with teachers and learners including the support activities and outcome measures.

The NEP was implemented as summarized in Figure 1. The treatment school teachers were trained to use the contextual NE materials in a 1 day workshop at the end of the 2014 school year. Though all the teachers of LS and NST in Grades 4–7 were included in the training in order to strengthen support for the NEP, only the LS and NST teachers of Grades 5 and 6 were needed to use the NE materials. Each learner received a copy of the contextual NE workbook with which they participated in the nutrition lessons. Learners who did not provide parental consent or who did not assent to participate in the study were not exempted from the nutrition lessons. However, they were excluded from data collection. The control school teachers and learners did not receive any of the developed NE materials. The teachers taught nutrition in the LS and NST subjects in the usual manner.

The developed NE materials were implemented in the allotted periods and time set out for different topics in the DoBE curriculum, starting in February and ending in October 2015. For Grade 6, nutrition topics were taught for 5 weeks in February and March in 32 classes and for 2 weeks in October in nine classes. For Grade 5, nutrition topics were taught for 2 weeks in July and August in six classes. The duration of each class was 30 minutes. Prior to teaching the nutrition topics, we conducted a re-orientation session with all the teachers of LS and NST in Grades 5 and 6 to refresh their understanding on how to use the manual. While the topics were being taught, we visited the school to supply foods and materials for class demonstrations. We contacted the teachers telephonically to receive their feedback as implementation progressed. In total, teachers taught five nutrition topics; one teacher taught NST in Grade 6, two teachers taught LS in Grade 5, and three teachers taught LS in Grade 6. The principal of the treatment school ensured an enabling atmosphere for the implementation.

### Data Collection Instruments and Procedures

Separate nutrition KAP questionnaires were developed for teachers and learners. The questionnaire for teachers was adapted from relevant sections of three standardized questionnaires (35–37). The adaptations included questions on vending machines being adjusted to include food vendors, a reduction in the number of questions on fat, and inclusion of foods commonly eaten in South Africa. The section on the recommended five servings of fruits and vegetables a day was adjusted to reflect the guideline for vegetables and fruit intake in the South African Food-Based Dietary Guidelines (SAFBDGs) (38). The developed teacher's questionnaire comprised 53 knowledge items in five categories: current dietary recommendations for children; sources of nutrients; diet-disease relationship; food processing; and food hygiene. The knowledge questions solicited responses such as true, false or uncertain; yes or no; high or low. The attitude items comprised 12 Likert type questions with the options of “agree,” “do not agree,” or “not sure.” The practice items were 36 Likert type questions in four broad areas: personal dietary habits; eating habits at school; classroom food practices; and school-wide food practices. The Cronbach's alpha reliability coefficients for the internal consistency of the teacher's instrument were 0.9, 0.7, and 0.9 for the knowledge, attitude and practice items, respectively.

The nutrition KAP questionnaire for the learners was adapted from the previously validated questionnaire used in the Healthy and Effective Lifestyle in Children (HELIC) study (35). The questionnaire was adjusted to exclude questions on the food pyramid, and to include the SAFBDGs (38) and foods commonly eaten in South Africa. Some terms were changed to be familiar to South African learners, e.g., break instead of recess and cold drinks instead of carbonated drinks. The adapted learner's questionnaire consisted of 23 knowledge questions in five categories: food, nutrients and functions, food and energy, nutrient deficiencies, food choices, and sources of nutrients. Nine practice and 11 attitude questions were included. The Cronbach's alpha coefficients for the learners' instrument were 0.6, 0.6, and 0.5 for the knowledge, attitude and practice items, respectively.

The questionnaires were administered in English at the treatment and control schools, using the same procedures both before and after implementation. We obtained demographic information of the teachers including age, gender, years of teaching experience, highest qualification, and employment status. The teachers' nutrition KAP questionnaire was self-administered in 30 minute working lunch sessions, during the long break in the staff common room. This method allowed us to clarify where necessary, and to collect questionnaires immediately after completion. The learners' KAP questionnaires were administered with the help of research assistants in the learners' classrooms during school hours, both before and after implementation of the NEP. The questions were read aloud to the learners who then indicated their answers on the questionnaire. This method facilitated a uniform understanding of the questions among learners (39).

### Statistical Analysis

The data were entered into Microsoft Excel spreadsheets in duplicate for comparison after which errors were rectified. The data were analyzed using Stata^®^ Statistical Software Release 10, 2007. The demographic variables of the participants were analyzed using the Fischer's exact or the Pearson's chi-squared tests and were described by numbers and percentages.

The nutrition KAP values were expressed in percentages to simplify comparison across categories. A correctly answered nutrition knowledge question was assigned the score of one and zero if not correctly answered. Likewise, a score of one was assigned to attitude statements with a response that was consistent with healthy eating, while a response that was contrary to healthy eating was assigned the score of zero. The practice questions for teachers were scored using the scoring system for Likert type data (40). Response options were summed under the different practice categories to a possible lowest (zero) and a maximum possible score of between 10 and 30. Questions addressing healthy and unhealthy practices in the same category were summed separately. The nine learners' practice questions were assigned scores as described by Shariff (35). The first three questions had four options, a score of one was given to the options consistent with healthy eating while the other options were given the score of zero. The last six questions had four options; almost every day, several times a week, occasionally and never. While a score of 1 was given to the options of almost every day and several times a week, a zero score was given to the options of occasionally and never.

The nutrition KAP data were analyzed and described by numbers and percentages and by means and standard deviations. All KAP scores were tested for normality. Data were normally distributed; Shapiro Wilk “W” values for all variables were >0.8.

Independent samples *t*-test was used to compare the pre-implementation scores between the schools. Random effects Generalized Least Squares (GLS) regression analysis was used to estimate the overall change in the mean scores for nutrition KAP between the schools from pre-implementation to post-implementation while accounting for missing scores at post-implementation. The within school differences in the nutrition KAP from pre- to post-implementation followed from the same random effects GLS regression analysis. The choice of random effects GLS regression was because of its capacity to estimate changes between and within measures in a single model while accounting for missing data. Also, when validated against the more frequently used likelihood ratio test, the random effects GLS regression was found to be effective in handling unbalanced data and sample designs of as small as 15 (41, 42). The level of significance for nutrition KAP was set at 0.025 for a one-sided test since we hypothesized a significant improvement in the nutrition KAP of the treatment school teachers and learners over that of the control school teachers and learners. That of the demographic variables was set at 0.05 for a two-sided test since we were comparing means and frequency distributions of the two schools.

## Results

### Participants' Characteristics

**Figure 2:**
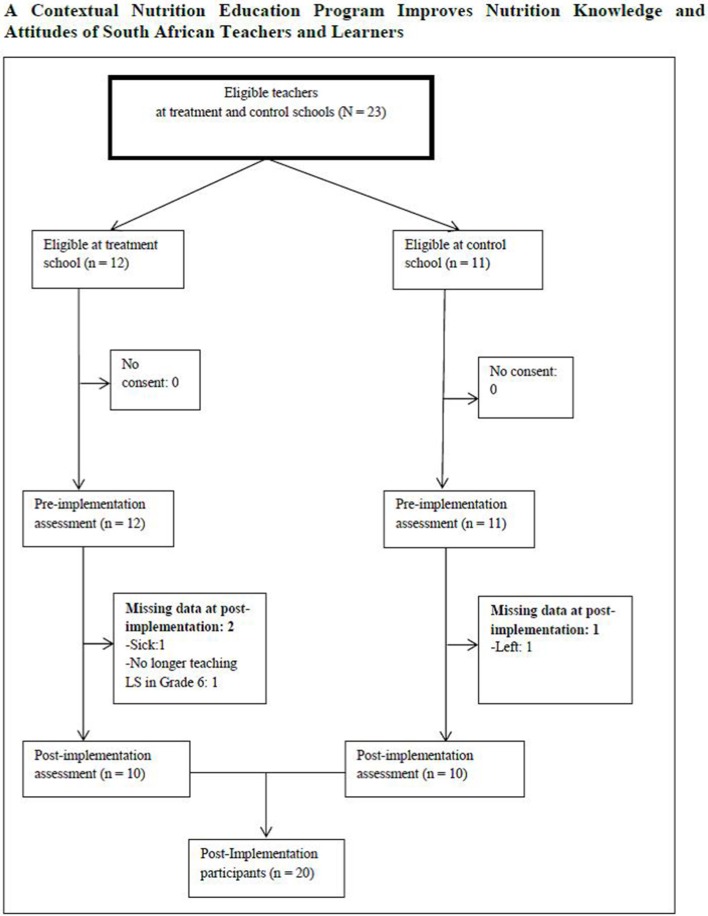
Flow of teachers who participated in the nutrition education program in two primary schools in bronkhorstspruit.

**Figure 3:**
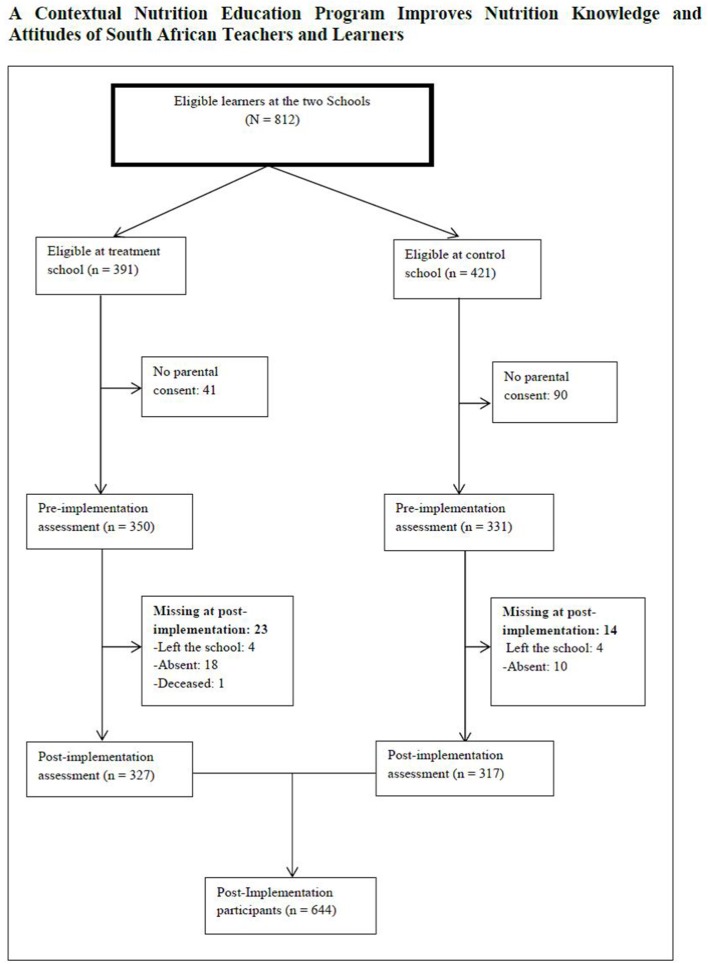
Flow of learners who participated in the nutrition education program in two primary schools in bronkhorstspruit.

Twenty three teachers (12 in the treatment school) participated in the study. There were three dropouts at post-implementation (Figure 2). The mean age of the teachers was 46 ± 7.9 years with 18 ± 9.7 mean years of teaching experience. Most of the teachers (82.5%) were employed full time and were women (60.9%). Nearly half (47.8%) had a teaching diploma as their highest educational qualification. A total of 681 learners (Grades 5 and 6) in the treatment (*n* = 350) and control (*n* = 331) schools participated in the study as shown in the flow of the participating learners (Figure 3). The mean age of the Grade 5 learners was 10.5 ± 1.2 years, while that of Grade 6 learners was 11.6 ± 1.0 years. There were 338 male learners. The learners were primarily of the black race (98.9%), and the remainder were colored (people of mixed decent including Khoisan, African, Malay, Chinese, and white). Most of the learners (91%) had heard about healthy eating from teachers (38%) and doctors or nurses (26%) in the treatment school, and from family members (33%) in the control school.

The number of dropouts had no effect on the demographic variables of teachers and learners at post-implementation (*p* ≥ 0.05). Likewise, there was no difference in the distribution of demographic variables from pre- to post-implementation.

### Teachers' Nutrition Knowledge

The mean nutrition knowledge scores at pre- and post-implementation for the treatment and control schools are shown in Table 1. The difference in the total nutrition knowledge scores between the treatment and control school from pre- to post-implementation was significant (*p* = 0.003). The treatment school had a higher total mean score of 85.5% ± 8.2. The within school difference was greater in the treatment school which showed a mean change of 14.1% in comparison to the control school with a mean change of 0.9%. Regarding the categories of nutrition knowledge, the treatment school had higher mean scores for all the categories, but only the difference for the sources of nutrients category (86.1% ± 10.2 against 69.6% ± 13.1, *p* ≤ 0.001) was significant. While the treatment school showed a within school improvement of 14.3% for this category, the control school showed a decrease of −1.6% (see Table 1).

**Table 1:** Comparison of the nutrition knowledge, attitudes and practices scores of the teachers at pre- and post-implementation (*N* = 23).

**Schools/variables**	**Pre-implementation**			**Post-implementation**						**^4^*p*-value**
	**Treatment school score (%) Mean ± SD (*n* = 12)**	**Control school (Score %) Mean ± SD (*n* = 11)**	**^1^*p* value**	**Treatment school (*****n*** **=** **10)**			**Control school (*****n*** **=10)**			
				**Score (%) Mean ± SD**	**^2^Mean change (%)**	**^3^*p*-value**	**Score (%) Mean ± SD**	**^2^Mean change (%)**	**^3^*p*-value**	
**Nutrition knowledge**										
Total nutrition knowledge	71.3 ± 8.2	71.5 ± 10.4	0.051	85.5 ± 8.2	14.1	<0.001	73.4 ± 10.3	0.9	0.786	0.003
Current dietary recommendations for children	75.0 ± 15.0	77.3 ± 21.5	0.297	91.3 ± 8.4	16.2	0.002	88.8 ± 9.2	10.7	0.116	0.59
Sources of nutrients	71.1 ± 9.9	70.1 ± 9.9	0.242	86.1 ± 10.2	14.3	<0.001	69.6 ± 13.1	−1.6	0.653	<0.001
Diet disease relationship	69.8 ± 17.2	69.3 ± 15.1	0.074	83.8 ± 13.2	14	0.036	71.3 ± 21.3	2.2	0.713	0.19
Food processing	70.0 ± 23.4	70.9 ± 18.7	0.919	74.0 ± 23.2	4	0.594	68.0 ± 19.3	−2.9	0.726	0.44
Food hygiene	70.8 ± 17.9	75.0 ± 19.4	0.538	87.5 ± 17.7	17.8	0.005	80.0 ± 19.7	5	0.558	0.24
**Attitudes**	73.6 ± 20.0	71.9 ± 14.1	0.237	84.2 ± 16.4	9.1	0.093	75.8 ± 17.3	4.2	0.479	0.531
**Practices**										
Healthy personal dietary practices	70.8 ± 19.0	51.2 ± 17.8	0.019	70.3 ± 15.7	0.4	0.93	59.7 ± 14.4	7.9	0.19	0.34
Unhealthy personal dietary practices	40.0 ± 20.9	30.9 ± 18.6	0.282	45.6 ± 12.5	3.4	0.51	39.6 ± 23.7	8.3	0.22	0.17
Healthy dietary practices at school	51.7 ± 34.3	63.6 ± 28.0	0.371	73.0 ± 22.1	21.4	0.17	53.0 ± 34.3	−10.7	0.35	0.52
Unhealthy dietary practices at school	62.5 ± 24.1	67.3 ± 21.0	0.615	76.0 ± 8.4	14.2	0.09	55.0 ± 35.4	−12.7	0.18	0.87
Classroom food practices	24.3 ± 15.7	21.9 ± 20.2	0.755	19.2 ± 18.5	−4.7	0.36	15.8 ± 12.7	−6.0	0.37	0.20
Healthy school wide food practices	81.9 ± 15.2	83.8 ± 11.5	0.737	83.9 ± 10.3	2.9	0.43	85.0 ± 16.1	1.2	0.84	0.55
Unhealthy school wide food practices	38.9 ± 21.7	21.2 ± 15.1	0.034	31.7 ± 22.5	−6.5	0.36	23.3 ± 22.8	1.7	0.85	0.67
Practices in food hygiene	22.9 ± 14.9	27.3 ± 15.6	0.498	23.8 ± 17.1	0.8	0.91	25.0 ± 10.2	−2.3	0.58	0.88

1 *Difference between schools derived from independent samples t-test*.

2 *Discrete change from the base level derived from random effects GLS regression*.

3 *Within school difference derived from random effects GLS regression*.

4 *Difference between the mean changes between schools from pre- to post-implementation derived from random effects GLS regression*.

### Teachers' Nutrition Attitudes

Regarding the nutrition attitudes of the teachers, the treatment school had a higher mean score (84.2% ± 16.4) than the control school (75.8% ± 17.3) at post-implementation. However, the difference was not significant (*p* = 0.531). The within school difference was 9.1 and 4.2% for the treatment and control schools, respectively.

### Teachers' Dietary Practices

The dietary practices were assessed in eight categories (Table 1): Healthy personal dietary practices; unhealthy personal dietary practices; healthy dietary practices at school; unhealthy dietary practices at school; classroom food practices; healthy school-wide food practices; unhealthy school-wide food practices; and practices in food hygiene. There were no significant differences for all the categories of dietary practices (*p* ≥ 0.025). The mean scores ranged from 19.2% ± 18.5 to 83.9% ± 10.3 in the treatment school, and from 15.8% ± 12.7 to 85.0% ± 16.1 in the control school at post-implementation for all the categories. In the treatment school, there were within school increases in the mean score for the categories on healthy personal dietary practices, unhealthy personal dietary practices, healthy dietary practices at school, unhealthy dietary practices at school, healthy school-wide food practices, and practices in food hygiene. However, the increases were not significant (*p* ≥ 0.025).

### Learners' Nutrition Knowledge

The mean total nutrition knowledge scores for the learners at pre- and post-implementation are shown in Table 2. The treatment school had a significantly higher mean score than the control school (53.2% ± 16.9 against 53.1% ± 17.6, *p* = 0.001) at post-implementation. The treatment school showed a significant within school improvement of 4.9% (*p* < 0.001) in comparison to a slight decline (−0.1%, *p* = 0.96) within the control school. The mean scores for the categories of nutrition knowledge showed that the treatment school had significantly higher mean scores than the control school for the categories of food and energy (*p* < 0.001), nutrient deficiency (*p* = 0.008) and sources of nutrients (*p* = 0.002). The treatment school also had significant within school improvement for all the categories of nutrition knowledge except for the category of food, nutrients, and functions. The control school had no significant within school improvements in any of the categories of nutrition knowledge (*p* ≥ 0.025).

**Table 2:** Comparison of the nutrition knowledge, attitudes, and practices scores of the learners at pre- and post-implementation (*N* = 681).

**Schools/variables**	**Pre-implementation**			**Post-implementation**						**^4^*p*-value**
	**Treatment school Score (%) Mean ± SD (*n* = 350)**		**Control school (% Score) Mean ± SD (*n* = 331)**		***^1^p*-value**	**Treatment school (*****n*** **=** **327)**			**Control school (*****n*** **=** **317)**	
		**Score (%) Mean ± SD**	**^2^Mean change (%)**	**^3^*p*-value**	**Score (%) Mean ± SD**	**^2^Mean change (%)**	**^3^*p*-value**			
**Nutrition knowledge**										
Total nutrition knowledge	48.3 ± 14.5	53.2 ± 16.7	<0.001	53.2 ± 16.9	4.9	<0.001	53.1 ± 17.6	−0.1	0.96	0.001
Food nutrients and functions	48.8 ± 24.5	52.7 ± 26.9	0.048	46.6 ± 22.1	−2.2	0.222	53.4 ± 23.4	0.8	0.558	0.192
Food and energy	68.3 ± 29.5	70.6 ± 31.2	0.323	77.9 ± 26.5	9.5	<0.001	67.6 ± 30.6	−2.9	0.168	<0.001
Nutrient deficiency	55.5 ± 33.1	63.5 ± 32.8	0.001	62.0 ± 32.3	6.4	0.009	61.2 ± 33.8	−2.4	0.275	0.008
Food choices	57.1 ± 24.4	66.1 ± 26.6	<0.001	63.4 ± 26.7	6.3	0.001	67.6 ± 27.9	1.7	0.336	0.073
Sources of nutrients	30.0 ± 17.0	32.5 ± 17.8	0.061	36.4 ± 22.6	6.4	<0.001	32.8 ± 18.7	0.3	0.813	0.002
**Attitudes**	56.9 ± 18.0	55.7 ± 19.4	0.403	63.9 ± 19.7	6.9	<0.001	56.8 ± 19.6	1.1	0.419	0.002
**Practices**	63.1 ± 16.9	62.3 ± 17.5	0.544	60.0 ± 19.7	−2.5	0.039	62.2 ± 16.8	−0.1	0.93	0.24

1 *Difference between schools derived from independent samples t-test*.

2 *Discrete change from the base level derived from random effects GLS regression*.

3 *Within school difference derived from random effects GLS regression*.

4 *Difference between the mean changes between schools from pre- to post-implementation derived from random effects GLS regression*.

### Learners' Nutrition Attitudes

The mean scores of the learners' nutrition attitudes in the two schools are shown in Table 2. At post-implementation, the treatment school had a significantly higher mean score than the control school (63.9% ± 19.7 against 56.8% ± 19.6, *p* = 0.002). The treatment school had a significant within school improvement with a mean change of 6.9% (*p* < 0.001), while the control school had a non-significant within school improvement with a mean change of 1.1% (*p* = 0.419).

### Learners' Dietary Practices

Estimating the differences in the mean scores between the two schools from pre- to post-implementation indicated no significant difference (*p* = 0.24) (see Table 1). The within school differences in the dietary practices from pre- to post-implementation indicated a decrease in both the treatment and the control schools with a mean change of −2.5 and −0.1%, respectively. However, the negative trends were not significant (*p* = 0.039 and *p* = 0.93, respectively).

## Discussion

The present study tested the hypothesis that the contextual NEP would lead to a significant improvement in the nutrition KAP of the treatment school teachers and learners compared to the control school. The teachers in the treatment school significantly improved (*p* = 0.003) in nutrition knowledge compared with the teachers in the control school. This result was substantiated by previous studies where NE intervention led to a significant improvement in the nutrition knowledge of teachers in the intervention group (24, 43–45).

The improvement in the nutrition knowledge of the treatment school teachers could be explained by the formal exposure of the teachers during training and implementation. The teachers received training on the use of NE materials in the teaching of nutrition topics, and revised the nutrition topics before teaching the lessons. Furthermore, teachers had to prepare before teaching the lessons and hence improved their own knowledge. A study which explored the relationship between resources for teaching nutrition and nutrition knowledge found that the nutrition knowledge of the teachers was positively associated with the nutrition lessons they taught (45). In this study, teachers in the treatment schools scored exceptionally well on the category of sources of nutrients (Table 1), which had the highest number of knowledge questions (28 out of 53) and comprised questions covering areas such as carbohydrate, fat, protein, and vitamin sources.

Within school differences showed that the treatment school teachers significantly improved in the categories of dietary recommendations for children (*p* = 0.002), sources of nutrients (*p* < 0.001), and food hygiene (*p* = 0.005) (Table 1). The significant improvement in the category of food hygiene was in spite of the fact that they had not yet taught the topic at the time of the assessment. However, the teachers had been exposed to the topic prior to the intervention during the 1 day workshop, where they had been trained in the use of the NE manual. The control school teachers had non-significant improvements in the categories of current dietary recommendations for children, diet-disease relationship, and food hygiene.

In respect to nutrition attitudes, the treatment school teachers improved compared to the control school teachers (post-implementation mean scores of 84.2% ± 16.4 and 75.8% ± 17.3 for treatment and control schools, respectively). However, the difference was not significant (*p* = 0.531). A non-significant within school improvement with a mean change of 9.1% was observed with the treatment school while the control school improved attitudes by 4.1%. A study that assessed the nutrition attitudes of child care teachers found an average score of 83% and the older teachers had more desirable attitudes (46). The findings regarding the nutrition attitudes of the treatment school teachers are noteworthy even through the improvement compared to control school teachers was not significant. Teachers who have positive attitudes may be more supportive of NE in schools (47). Moreover, the teachers in the present study, being of the middle age group (mean age 46 ±7.9 years), may be more inclined to value healthy eating as a result of life experiences as shown by Choi (46).

The treatment school teachers did not significantly improve in comparison to the control school teachers in any of the categories of dietary practices assessed. The teachers' personal dietary practices in both schools depicted both healthy and unhealthy eating to varying degrees. However, a non-significant within school improvement was recorded in six of the eight categories of dietary practices for the treatment school and in five of the eight categories for the control school. A survey of the dietary variety of South Africans revealed a generally low score particularly among black South Africans, with vitamin A rich fruits and vegetables and legumes and nuts being the most neglected food groups (48). The low score in respect of dietary diversity among the black South Africans reflected the dietary practices of the teachers in this study. However, low consumption of fruits and vegetables is not limited to the South African population. Studies outside of South Africa found quite low fruit and vegetable intake and high fried food intake among teachers studied (49–51).

Compared to learners in the control school, learners in the treatment school had a significantly higher total mean post-implementation nutrition knowledge score (*p* = 0.001). A study that used the same instrument as the present study also reported a significant improvement in nutrition knowledge (*F* = 17.72, *p* < 0.001) (35). Similar results were reported by previous South African studies on school NE interventions. A significant improvement was observed in the nutrition knowledge of Grade 7 learners with a change in score from 45 to 58%, *p* ≤ 0.001 (23). Also, De Villiers and colleagues reported a significant improvement in learners' nutrition knowledge with a mean difference of 1.88, *p* = 0.021 (52).

Our results might be explained by a number of reasons, including a participatory teaching approach, which promotes active and interactive learning (16, 53). Each learner received a learner's workbook that enhanced their participation in class discussions. School-based NE accompanied by education materials such as posters, workbooks, and games have been reported to lead to improvement in learners' nutrition knowledge (54, 55). The practical and class demonstrations provided opportunities for experiential learning, which is known to contribute to improving the nutrition knowledge of primary school learners (1).

The treatment school learners had significant within school improvement in all the categories of nutrition knowledge (*p* < 0.001, *p* = 0.009, *p* = 0.001, and *p* < 0.001), except in the category of food, nutrients, and functions. The learners in the control school had non-significant within school improvements in the categories of food, nutrients, and functions (*p* = 0.558), food choices (*p* = 0.336), and sources of nutrients (*p* = 0.813), and had a decline performance in the categories of food and energy, and nutrient deficiency. It should be noted that despite a significant within group improvement (for treatment school) in the category of the sources of nutrients the mean score remained very low (from 30.0% ± 17.0 to 36.4% ± 22.6 for treatment school and 32.5% ± 17.8 to 32.8% ± 18.7 for control school, pre- to post-implementation). This very low score contributed to the low total mean scores of 53.2% ± 16.9 and 53.1% ± 17.6 for the treatment and control schools, respectively. The low mean score for the category of the sources of nutrients in both schools could have implications for the learners' skill in identifying healthy foods. A study that investigated the reasons children make their food choices reported that the children's view about what foods were healthy was inadequate (56).

Considering the nutrition attitudes of the learners, the learners in the treatment school significantly improved (*p* = 0.002) compared with the learners in the control school. There was also a significant within school improvement (*p* ≤ 0.001). This result upholds the findings of Shariff and colleagues who used the same instrument and reported a significant change (*F* = 6.41, *P* < 0.05) in the attitudes of the learners (35). The reason for the significant improvement in the present study might be connected with the way the nutrition topics were presented and the improvement in the learners' nutrition knowledge. The aim of NE is essentially to improve nutrition knowledge toward enhancing nutrition attitudes and behaviors (57).

The dietary practices of the learners in the treatment school did not significantly improve in comparison with the learners in the control school (*p* = 0.24). The non-significant decline in the learners' dietary practices in the treatment school in the present study is notwithstanding the significant improvement in the nutrition knowledge and attitudes. The resource-constrained environment of the learners, and the fact that the learners had no control over what they ate in their homes, might explain the result in this present study. Gorely and colleagues explained how parental influence plays a role in a non-significant improvement observed in the learners' intake of fruits and vegetables (58). The results of the present study, along with the findings of previous studies in the literature, indicate that improvement in nutrition knowledge does not always translate to healthy dietary practices (58, 59). These results corroborate the findings of previous studies that led to significant improvements in the nutrition knowledge and self-efficacy of primary school children, but with no improvement in their eating behavior in the intervention or control schools (9, 25). Likewise, the intervention by Duncan and colleagues reported significant improvements in the other intervention outcomes, but did not achieve a significant effect on the learners' consumption of unhealthy drinks (60). It is documented that a significant positive impact on the dietary practices is seldom realized with short intervention durations (e.g., <1 year) (57, 61). The short duration of the NEP in the present study might have been insufficient to establish a change in the dietary practices of the learners. The learners were only exposed to the intervention in segments as scheduled by the DoBE curriculum during the 2015 school year.

An important strength of this study was that the NEP was delivered by teachers trained in nutrition and, topics were implemented in line with the content and allocated teaching periods in the DoBE curriculum. As a result, the teachers implemented the NEP without compromising their official teaching duties. This approach won the support of the principal of the treatment school, confirming the finding that aligning a school NE initiative with the school curriculum increases support from school staff (60). Implementation by the teachers could facilitate the sustainability of the NEP, such that the continuous external input of experts would not be needed.

The limited number of schools involved in the study and the implementation approach made it impossible to use a randomized controlled trial (RCT) which is considered a gold standard in evaluation. The study was also limited in the number of teachers who participated in the study. Including a larger number of teachers and more schools could have led to a better assessment of the impact of the NEP. The fact that the study was conducted in one district may limit the generalizability of the results to other districts in South Africa. The low Cronbach alpha values of 0.6, 0.6, and 0.5 for the nutrition knowledge, attitudes, and practices items in the learners' questionnaire might be an indication of a misunderstanding of some of the questions. However, the low number of questions in the questionnaire might have also influenced the values as explained by Tavakol et al. (62).

## Conclusion

The contextual NEP resulted in a significant improvement in the teachers' nutrition knowledge. The nutrition attitudes of the treatment school teachers did not significantly improve, though there was an appreciable within school increase and in the mean score compared with the control school teachers. The NEP did not improve the dietary practices of the teachers. The NEP resulted in a significant improvement in the learners' nutrition knowledge and nutrition attitudes in comparison with the control school and within school, but no improvement in the dietary practices was observed.

The findings from this present study demonstrated that implementing a contextual NEP for primary school teachers in resource-limited settings could lead to significant improvement in the teachers' and the learners' nutrition knowledge. The significant improvement in the nutrition knowledge of the learners is particularly beneficial in equipping the learners with nutrition knowledge which can be useful for them as adults. It has been advocated that NE for learners in a resource-limited setting should target nutrition knowledge acquisition as future parents (16).

In view of the aim of the DoBE that learners receive relevant knowledge to benefit their lives, it is recommended that the context-specific NE materials, developed and used in this study, be considered for use in teaching nutrition in the primary schools in Gauteng province, South Africa.

## Ethics Statement

This study was carried out in accordance with the recommendations and with the permission of the Gauteng Department of Education (Numbers: D2014/199; D2014/308A; and D2015/374A). Ethical approval was provided by the Research Ethics Committee of the Faculty of Natural and Agricultural Sciences of the University of Pretoria (Number: EC130424-037). All teachers and learners provided written informed consent and assent in accordance with the Declaration of Helsinki.

## Author Contributions

UM, GG, MK, and PB conceptualized the study. MK collected the data and wrote the first draft of the manuscript with assistance from UM and GG. Statistical analysis was performed by PB. All authors reviewed and approved the draft of the manuscript and ensured the accuracy and integrity of the work before submission to the journal.

### Conflict of Interest Statement

The authors declare that the research was conducted in the absence of any commercial or financial relationships that could be construed as a potential conflict of interest.
